# A SYSTEMATIC REVIEW ON AGING IN THE COMMUNITY AND CULTURE AMONG CHINESE OLDER ADULTS

**DOI:** 10.1093/geroni/igad104.1182

**Published:** 2023-12-21

**Authors:** Hao-Min Chen, Su-I Hou

**Affiliations:** Texas A&M University-Central Texas, Killeen, Texas, United States; School of Global Health Management & Informatics, University of Central Florida, Orlando, FL, USA, Oviedo, Florida, United States

## Abstract

Chinese older adults face many barriers to healthy aging in community, such as increased financial burden, threatened place security, limited accessibility to medical services, language and transportation difficulties, daily housework and repairs challenges, etc. This systematic review aimed to examine and report the results current scholars have published in journal articles in the last decade (2010-present) on the influence of culture on Chinese older adults aging in community. Combination terms of “aging in community or aging in place,” “Chinese,” and “culture or cultural or ethnicity or identity or values” were used when searching in the four databases: CINAHL Complete; ERIC; MEDLINE; and PsycInfo. Researcher examined abstracts of all journal articles retrieved using the above criteria and further determined their relevance for inclusion. A total of 38 journal articles were included. Findings suggest that aging in community reflects a changing policy tendency from institution-based to community-centered care which better responds to the needs of the growing Chinese senior population. Family care remains older adults’ preferences. However, individual, community, and larger contextual factors can be influenced, such as the number of adult children in the household, education level, residential area, accessibility to healthcare facilities, perceived cultural values of filial piety, state-owned enterprise reformation, and the one-child policy in China. Policymakers are encouraged to promote older adults’ personalized care plans; comprehensive health insurance coverage; constructive social/community environments (e.g. community parks or community open spaces for age-friendly activities); home care support, or smart home technologies for regular health monitoring.

